# Amyloid and tau cerebrospinal fluid biomarkers in HIV infection

**DOI:** 10.1186/1471-2377-9-63

**Published:** 2009-12-22

**Authors:** Magnus Gisslén, Jan Krut, Ulf Andreasson, Kaj Blennow, Paola Cinque, Bruce J Brew, Serena Spudich, Lars Hagberg, Lars Rosengren, Richard W Price, Henrik Zetterberg

**Affiliations:** 1 Department of Infectious Diseases, University of Gothenburg, Sahlgrenska University Hospital, SE-416 85 Gothenburg, Sweden; 2 Department of Psychiatry and Neurochemistry, University of Gothenburg, Sahlgrenska University Hospital, Gothenburg, Sweden; 3 Clinic of Infectious Diseases, San Raffaele Hospital, Milan, Italy; 4 Departments of Neurology and HIV Medicine, St. Vincent's Hospital, University of New South Wales, Sydney, Australia; 5 Department of Neurology, University of California, San Francisco General Hospital, CA, USA

## Abstract

**Background:**

Because of the emerging intersections of HIV infection and Alzheimer's disease, we examined cerebrospinal fluid (CSF) biomarkers related of amyloid and tau metabolism in HIV-infected patients.

**Methods:**

In this cross-sectional study we measured soluble amyloid precursor proteins alpha and beta (sAPPα and sAPPβ), amyloid beta fragment 1-42 (Aβ_1-42_), and total and hyperphosphorylated tau (t-tau and p-tau) in CSF of 86 HIV-infected (HIV+) subjects, including 21 with AIDS dementia complex (ADC), 25 with central nervous system (CNS) opportunistic infections and 40 without neurological symptoms and signs. We also measured these CSF biomarkers in 64 uninfected (HIV-) subjects, including 21 with Alzheimer's disease, and both younger and older controls without neurological disease.

**Results:**

CSF sAPPα and sAPPβ concentrations were highly correlated and reduced in patients with ADC and opportunistic infections compared to the other groups. The opportunistic infection group but not the ADC patients had lower CSF Aβ_1-42 _in comparison to the other HIV+ subjects. CSF t-tau levels were high in some ADC patients, but did not differ significantly from the HIV+ neuroasymptomatic group, while CSF p-tau was not increased in any of the HIV+ groups. Together, CSF amyloid and tau markers segregated the ADC patients from both HIV+ and HIV- neuroasymptomatics and from Alzheimer's disease patients, but not from those with opportunistic infections.

**Conclusions:**

Parallel reductions of CSF sAPPα and sAPPβ in ADC and CNS opportunistic infections suggest an effect of CNS immune activation or inflammation on neuronal amyloid synthesis or processing. Elevation of CSF t-tau in some ADC and CNS infection patients without concomitant increase in p-tau indicates neural injury without preferential accumulation of hyperphosphorylated tau as found in Alzheimer's disease. These biomarker changes define pathogenetic pathways to brain injury in ADC that differ from those of Alzheimer's disease.

## Background

Central nervous system (CNS) infection is a nearly uniform feature of untreated human immunodeficiency virus type 1 (henceforth, *HIV*) infection. Thus, from initial viremia until death, HIV is detected in the cerebrospinal fluid (CSF) of most patients not treated with combination antiretroviral therapy [1-4]. While in its chronic phase CNS infection is usually unaccompanied by neurological symptoms or signs, this seemingly innocent exposure can give way to more 'invasive' HIV encephalitis (HIVE) that manifests clinically as the AIDS dementia complex (ADC; also termed HIV-associated dementia), most commonly in the context of more advanced systemic infection [5,6]. The salutary effects of potent antiretroviral therapy that have transformed systemic infection from an almost invariably fatal condition into a chronic disease amenable to medical management with prolonged survival have also had a major impact on its CNS manifestations. This includes not only a marked reduction in the incidence of CNS opportunistic infections, but a similar decline in ADC/HIVE [7]. The latter parallels the potent effects of antiretroviral therapy on CSF HIV RNA concentrations [8-12].

The longer lifespan of patients on antiretroviral treatment has raised questions of whether HIV infection might interact with or even potentiate the development of Alzheimer's disease [13-15]. Indeed, the pathogenesis of HIV-related brain injury may intersect with Alzheimer's disease in several aspects. Thus, some reports suggest that brain amyloid deposition is increased in HIV infection, though the extent and relation of this deposition to the clinical state and regional HIV infection remain controversial [16-19]. A CSF biomarker pattern similar to that in Alzheimer's disease, with decreased CSF amyloid beta 1-42 fragment (Aβ_1-42_) and increased CSF total tau (t-tau) [20,21], has been reported in patients with ADC in one study [22]. Reports of elevated CSF t-tau and hyperphosphorylated tau (p-tau) in ADC are conflicting, and while some previous studies have shown increased CSF levels in patients with ADC [22,23], others have not [24,25].

In order to more clearly characterize changes in CSF biomarkers in HIV infection related to CNS amyloid and tau metabolism that are salient features of Alzheimer's disease, we measured concentrations of two forms of soluble amyloid precursor protein, alpha and beta (sAPPα and sAPPβ), together with Aβ_1-42_, t-tau and p-tau in CSF of untreated HIV-infected (HIV+) subjects with and without ADC, along with HIV+ subjects suffering CNS opportunistic infections and HIV seronegative (HIV-) control groups that included Alzheimer's disease patients and both younger and older controls. Specifically, this study tests the hypothesis that HIV infection induces a neurodegenerative process that from a biomarker perspective is distinct from Alzheimer's disease.

## Methods

### Study design

This was a cross-sectional study using archived CSF specimens from three clinical centers in Gothenburg, Sweden, Milan, Italy and San Francisco, USA. The HIV+ subjects selected included neuroasymptomatic, ADC and opportunistic infection groups who were either naïve to or off antiretroviral treatment for at least 6 months at the time of sampling. The HIV- subjects included younger controls age-matched to the HIV+ subjects and older subjects with or without Alzheimer's disease. Diagnoses of ADC and each of the opportunistic infections were based on CDC and American Academy of Neurology AIDS Task Force criteria using standard clinical and laboratory evaluations [26-28]. For CMV encephalitis and PML diagnosis, CMV or JC virus DNA, respectively, was detected in CSF [29,30]. ADC was staged according to the Memorial Sloan-Kettering scale [5]. The diagnosis of Alzheimer's disease conformed to the National Institute of Neurological and Communicative Disorders and Stroke - Alzheimer's Disease and Related Disorders Association (NINCDS-ADRDA) criteria [31].

CSF samples were obtained under the auspices of research protocols approved by the institutional review boards of each of the study sites and in accord with the Helsinki Declaration, either within the context of studies of the natural history of HIV infection or during diagnostic evaluations [10]. All subjects gave their informed consent, and if their capacity to provide consent was questioned, consent was also obtained from those with power of attorney.

### CSF and blood measurements

For the neural biomarker and HIV RNA assays, CSF was collected and processed in polypropylene tubes, centrifuged after collection to remove cells, and supernatants aliquoted and stored at -70°C until the time of the assay. CSF concentrations of sAPPα and sAPPβ were determined using the MSD^® ^sAPPα/sAPPβ Multiplex Assay as described by the manufacturer (Meso Scale Discovery, Gaithersburg, MD, USA). The samples were analyzed for Aβ_1-42_, t-tau, and p-tau (phosphorylated at threonine 181) by enzyme-linked immunosorbent assay (ELISA), as previously described [32-34]. All samples were analyzed together in batch at the University of Gothenburg using the same batches of reagents. Intra-assay coefficients of variation were below 10% for all analyses. Previously established laboratory cutoffs for Aβ_1-42_, t-tau and p-tau were used for descriptive analysis.

HIV-1 RNA was quantified by the Roche Amplicor Monitor assay (version 1.0 and 1.5, Hoffman La-Roche, Basel, Switzerland). Routine assessments also included CSF white blood cell (WBC) count and peripheral blood CD4+ T-lymphocyte (CD4) cell determination performed in the local clinical laboratories using routine methods.

### Statistical methods

Statistical significance of differences between groups was assessed using either ANOVA with Tukey's *post hoc *test for multiple comparisons or the Kruskal-Wallis test with Dunn's *post hoc *test. Statistical tests were conducted both with and without age adjustment. Spearman's Rank Correlation Coefficient was used to evaluate correlations among CSF variables, and linear regressions with 95% confidence intervals were used in graphing selected interactions. Receiver-operator characteristic (ROC) curve analysis of diagnostic sensitivity and specificity was performed for single variables. All these analyses used Prism 5 (Graphpad Software Inc, La Jolla, CA). Multivariate analysis used the Principal Component Analysis (PCA) algorithm implemented in the SIMCA-P+ software version 11.0.0.0 (Umetrics AB, Umeå, Sweden) [35].

## Results

### Subjects

The background subject characteristics are summarized in Table 1. They included: 40 HIV-infected without ADC referred to as *neuroasymptomatic *subjects who were either wholly without neurological symptoms or signs or showed only mild neurological signs attributed to static CNS conditions, all from San Francisco; 21 with a diagnosis of ADC (3 ADC stage 1 and 18 stage 2 - 4), all from Milan except two from San Francisco; and 25 with major CNS opportunistic infections - 4 with cytomegalovirus encephalitis (CMV-E), 6 cryptococcal meningitis, 6 cerebral toxoplasmosis and 9 progressive multifocal leucoencephalitis (PML) - all from Milan. The HIV- comparison groups included 22 younger subjects from San Francisco, 21 subjects with Alzheimer's disease from Gothenburg, and 21 older non-demented controls age-matched to the Alzheimer's disease group from Gothenburg. The ages were similar in the HIV+ groups and younger HIV- controls in which males also predominated, but differed from the Alzheimer's disease and older HIV- controls in which ages were again similar and there was a higher ratio of women. Among the HIV+ groups, the blood CD4+ T cell counts were lower in the ADC and OI groups than in the neuroasymptomatic group (P < 0.001 by post hoc testing) and the latter also, as expected, differed from the younger HIV- group (P < 0.01). While plasma HIV RNA levels were higher in the ADC and OI groups than the neuroasymptomatic group, this difference was not significant, though CSF HIV RNA levels were higher in the two neurologically afflicted groups compared to the neuroasymptomatic group (P < 0.001).

**Table 1:** Subject Characteristics.

	Number	Gender	Age	Blood CD4	Blood HIV	CSF HIV
			years	cells/μL	RNA copies/mL	
Subject Group		(F:M)	Mean (SD)		Median (IQR)	
***HIV Infected***						
Neuroasymptomatic (NA)	40	6:34	41 (7)	431 (96)	4.36 (3.50 - 4.85)	3.38 (2.34 - 3.79)
ADC	21	5:16	38 (8)	74 (96)	5.25 (4.76 - 5.50)	5.40 (5.19 - 5.50)
OIs	25	4:21	38 (9)	79 (87)	5.05 (4.92 - 5.66)	4.36 (3.95 - 5.00)

***HIV Uninfected***						
Cont y (HIV controls)	22	5:17	43 (7)	906 (265)	ND	ND
AD	21	12:9	65 (5)	ND	ND	ND
Cont o (AD controls)	21	9:12	68 (5)	ND	ND	ND

ND = not done

### Group differences in CSF biomarkers

**Figure 1:**
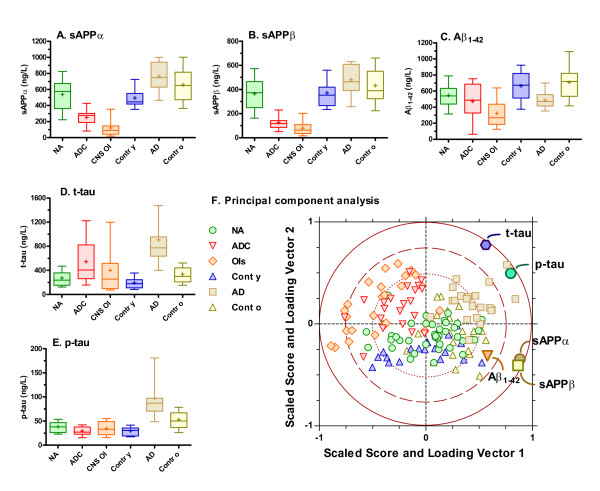
**Amyloid and tau CSF biomarker concentrations in the different patient groups and their interactions**. Panels A-E plot concentrations of different markers for the 6 subject groups. Boxes encompass interquartile ranges with median (line) and mean ("+"), while whiskers designate 10^th ^to 90^th ^percentiles. Results of statistical analysis of these data are given in Table 2A. Panel F shows the principal component analysis (PCA) score scatter plot of subjects identified by disease grouping with the superimposed variable loadings of the biomarkers. The data in Panels A - E were used to construct a two-dimensional model along the two vectors. The individual subjects are represented by the small symbols as indicated in the key, and their placement is determined by the combination of their marker values in the model. The placement of the five variables (large symbols as indicated) gives information about how each affects the placement of the individuals in the plot, both with respect to direction along each vector axis and the relative magnitude of this effect as indicated by their distance from the origin. For example, high p-tau and low sAPPβ values contribute importantly to the position in the upper right and left quadrants, respectively. Note, however, that all variables influence where each subject is located in the plot so that the plot provides a visual representation of how the subjects are separated by the results of all five biomarkers. For more detailed information on methods, see reference [35]. Abbreviations: NA, HIV+ neuroasymptomatics; ADC, AIDS dementia complex; CNS OIs, CNS opportunistic infections; Contr y, younger HIV- controls; AD, Alzheimer's disease; Contr o, older HIV- controls.

Figure 1, panels A - E, show the subject group results for each of the five CSF biomarkers (sAPPα, sAPPβ, Aβ42, t-tau, and p-tau). All showed significant intergroup differences (ANOVA P < 0.0001), though the particular subject group comparisons determining these differences varied as indicated by *post hoc *testing. Table 2 summarizes the statistical analysis of the clinically most relevant comparisons between the subject groups for each of the measured amyloid and tau metabolites. Age adjustment did not change any of the p-values, except that the statistical differences between younger and older controls disappeared.

**Table 2:** Comparisons of CSF neural biomarkers across groups.

	sAPPα	sAPPβ	Aβ_1-42_	t-tau	p-tau
**Overall ANOVA:**	**< 0.0001**	**< 0.0001**	**< 0.0001**	**< 0.0001**	**< 0.0001**

**Post Hoc Comparisons:**					
**ADC vs NA**	**< 0.001**	**< 0.001**	ns	**< 0.05**	ns
**OIs vs NA**	**< 0.001**	**< 0.001**	**< 0.001**	ns	ns
**ADC vs OIs**	ns	ns	ns	ns	ns
**NA vs Cont y**	ns	ns	ns	ns	ns

**AD vs Cont o**	ns	ns	**< 0.01**	**< 0.001**	**< 0.001**
**AD vs ADC**	**< 0.001**	**< 0.001**	ns	**< 0.01**	**< 0.001**

**Cont y vs Cont o**	**< 0.05**	ns	ns	ns	**< 0.01**

ns = not significant

* All results were also compared using non-parametric Kruskal-Wallis test that yielded similar results. Statistical tests were also conducted with age adjustment, which did not change any of the results reported above, except for that differences between younger and older controls disappeared.

#### Amyloid metabolites

While they differed in magnitude, CSF concentrations of sAPPα and sAPPβ (Figure 1A and 1B) exhibited similar patterns across the subject groups. Both were markedly reduced in the ADC and OI subjects compared to all the other groups, including the HIV+ neuroasymptomatics as well as the Alzheimer's disease patients and both HIV- control groups (P < 0.001 for all). CSF Aβ_1-42 _concentrations (Figure 1C) were less affected by HIV-related diseases: in the ADC group Aβ_1-42 _varied widely, and among the HIV+ subjects only the opportunistic infection group differed from the HIV+ neuroasymptomatics in this comparison. As expected, CSF Aβ_1-42 _concentrations in the Alzheimer's disease patients differed from the older controls (P < 0.01). However, CSF Aβ_1-42 _levels in the ADC patients differed from neither the infections nor the Alzheimer's disease groups.

#### Tau

CSF t-tau levels in the ADC group were elevated, but variable (Figure 1D), and differed from the neuroasymptomatics (P < 0.05) and younger HIV- controls (P < 0.001) and the Alzheimer's disease group (P < 0.01) which was higher, but not from the HIV+ opportunistic infections. Higher t-tau concentrations in the Alzheimer's disease group distinguished them from all the other groups except the ADC patients. P-tau concentrations (Figure 1E) did not differ among any of the HIV+ groups, nor did they differ from the younger HIV- controls, but these all differed from the Alzheimer's disease group which had the highest levels (P < 0.001). There was also an age effect, with the older HIV- controls having higher levels of p-tau than the younger controls (P < 0.01).

#### Principal component analysis

To assess how the combined effect of the five CSF biomarkers separated the subject groups, we applied principal component analysis to the data set shown in panels A - E of Figure 1. This analysis used all the groups and biomarker measurements and segregated the groups into three regions of the vector plot: one with the ADC and CNS OI subjects, another with the Alzheimer's disease subjects, and a third with the HIV+ neuroasymptomatics and HIV- groups (Figure 1, panel F). Thus, as anticipated by the similarity in the individual biomarker plots, the ADC and opportunistic infections clustered together in the left upper quadrant of the PCA plot. Likewise, the Alzheimer's disease subjects aggregated separately into the right upper quadrant, while the remaining subjects (neuroasymptomatic and HIV- control groups) all intermixed principally in the two lower quadrants. This analysis emphasizes the similarity of ADC and opportunistic infections in their effects on these neural markers, and their distinction from the Alzheimer's disease, on the one hand, and the other HIV+ and HIV- controls, on the other. The analysis of the effect of the five variables on the model (designated by the larger symbols in the Figure 1F plot) showed that the two sAPPs had the greatest influence in segregating the ADC and OI subjects, while Aβ_1-42 _exerted a smaller effect in the same direction. By contrast, t-tau and particularly p-tau were most important in the segregation of the Alzheimer's disease patients.

#### Opportunistic infections

**Figure 2:**
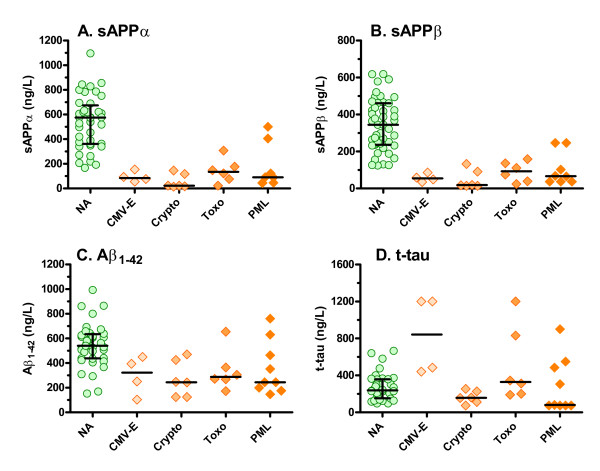
**CSF biomarkers in opportunistic infections**. The results for each of the biomarkers in the four infection subgroups are compared to the results of the HIV+ neuroasymptomatic subjects. The symbols show individual results while bars show medians and, for the neuroasymptomatics, the interquartile ranges. Abbreviations: NA, HIV+ neuroasymptomatics; CMV-E, CMV enecephalitis; Crypto, cryptococcal meningitis; Toxo, cerebral toxoplasmosis; PML, progressive multifocal leukoncephalopathy.

While the number of subjects with each of the four opportunistic infections was small, where the neural biomarkers were altered in the overall group, they were generally altered in the subgroups as well (Figure 2). Most notably, concentrations of both sAPPs were reduced in all four opportunistic infections in comparison to HIV+ neuroasymptomatic group (P ranging from < 0.05 to < 0.001), though there were no significant differences among the four different infections (Figure 2A and 2B). Reductions in CSF Aβ_1-42 _were also noted in all the opportunistic infections but were more variable, and only the cryptococcal meningitis group differed significantly from the neuroasymptomatic group (P < 0.05); again there were no differences among the four infections (Figure 2C). T-tau elevations (Figure 2D) were notable in individual patients in the CMV-E, toxoplasmosis and PML groups, but were also variable and none of the groups were statistically different from the neuroasymptomatic group. P-tau levels (not shown) were similar across all of the opportunistic infection groups, and none differed from the HIV+ neuroasymptomatics.

### Relationships among neural markers across subject groups

**Figure 3:**
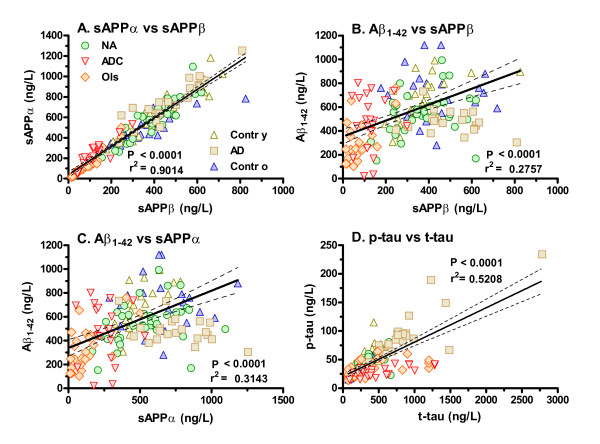
**Selected marker correlations across patient groups**. The four panels show relationships between markers: Panel A between sAPPα and sAPPβ, Panel B between Aβ_1-42 _and sAPPβ, Panel C between Aβ_1-42 _and sAPPα, and Panel D between p-tau and t-tau. Symbols for all four panels are defined in Panel A. The diagonal lines show linear regressions with 95% confidence intervals; each panel lists the P value and r^2 ^for the regression. Abbreviations are those given in Figure 1.

We also examined the correlations among the different biomarkers across the subject groups, with particular attention to the relationships within the amyloid pathway and between the tau forms. Initial exploration showed significant nonparametric correlations between the markers in the same pathways (sAPPα vs sAPPβ, P < 0.0001, r_s _= 0.957; sAPPα vs Aβ_1-42 _P = 0.06, r_s _= 0.153; sAPPß vs Aβ_1-42 _P < 0.05, r_s _= 0.195; t-tau vs p-tau P < 0.0001, r_s _= 0.462); sAPPα and sAPPβ also correlated with t-tau, P < 0.0001, r_s _= -0.644 and -0.736, and with p-tau P < 0.01 and P < 0.05, r_s _= 0.170 and 0.237. Figure 3A shows the high correlation of sAPPα and sAPPβ across the entire sample, indicating that the concentrations of these two metabolites were similarly altered by the different pathologies of the subjects. While Aβ_1-42 _also correlated with sAPPs across their range, the relationship was much looser. Figure 3B shows the variability within each clinical group in the CSF Aβ_1-42 _in relation to their CSF sAPPβ levels; this is perhaps most notable at low (abnormal) sAPPβ concentrations where the wide range of Aβ_1-42 _values was contributed by the ADC and opportunistic infection patients. Results were similar when Aβ_1-42 _was compared to sAPPα (Figure 3C). In Figure 3D showing the relationship of p-tau to t-tau concentrations, the different diagnoses influenced the regression in one or another direction - the elevated p-tau in the Alzheimer's disease patients with high t-tau pulled the right end of the line up, while the low p-tau in the ADC and infection patients with elevated t-tau pulled it in the opposite direction.

### Diagnostic utility of CSF amyloid and tau biomarkers for ADC

To define initial values that might be used as diagnostic 'cutoffs' for sAPP concentrations in ADC and to evaluate the utility of individual markers for ADC diagnosis in this series, we plotted receiver-operator characteristic (ROC) curves for each to compare their diagnostic sensitivity and specificity in relation to the other subjects. Table 3 summarizes results of analysis for each of the 5 biomarkers and their diagnostic utility in ADC diagnosis compared to all HIV+ subjects (neuroasymptomatic and opportunistic infections) and to the neuroasymptomatics alone (excluding the opportunistic infections). The reason for examining these two types of comparisons (with and without opportunistic infections) included: a) combined clinical presentation and neuroimaging usually separates the opportunistic infections from ADC before CSF is sampled, and this separation may be further enhanced by CSF identification of opportunistic pathogens (e.g., by CMV and JC virus DNA PCR and cryptococcal antigen); and b) it is clear from the individual marker data and principal component analysis in Figure 1 that these CSF biomarkers did not distinguish the two neurologically abnormal groups. This ROC curve analysis suggested initial diagnostic cutoffs optimizing both sensitivity and specificity of 333 ng/mL and 205 ng/mL for sAPPα and sAPPβ, respectively. The results also showed that both sAPPs, used individually might be diagnostically useful as shown. For sAPPβ both sensitivity and specificity were about 90 percent after excluding opportunistic infections, whereas specificity was lowered to less than 60 percent when the opportunistic infections were included. By contrast, both Aβ_1-42 _and t-tau were less helpful with relatively low sensitivity and specificity even after excluding the opportunistic infections. As anticipated from the similar p-tau levels in all the HIV+ groups, p-tau was not useful in establishing ADC diagnosis in the infected group because of its extremely low specificity. The relative utility of each of these markers for ADC was reflected in the areas under the concentration curve (AUC) values also listed in Table 2B, where a value of 1.00 represents perfect accuracy and 0.50 is no difference from random diagnosis. Thus, in this series, sAPPβ provided the most helpful marker of ADC after elimination of opportunistic infections.

**Table 3:** ROC Curve Analysis of ADC Diagnosis.

	sAPPα	sAPPβ	Aβ_1-42_	t-tau	p-tau
**Cutoff value:**	***< 333 ng/mL***	***< 205 ng/mL***	***< 450 ng/L***	***> 300 ng/L***	***< 60 ng/L***

**All HIV+ subjects**					
**Sensitivity**	**90,5**	**90,5**	**57,1**	**64,0**	**100,0**
**Specificity**	**56,3**	**58,4**	**47,7**	**61,3**	**4,6**
**ROC curve AUC**	**0,61**	**0,68**	**0,54**	**0,70**	**0,65**

**HIV+ excluding OIs**					
**Sensitivity**	**90,5**	**90,5**	**42,9**	**64,0**	**100,0**
**Specificity**	**85,0**	**90,0**	**72,5**	**67,6**	**5,0**
**ROC curve AUC**	**0,88**	**0,95**	**0,57**	**0,74**	**0,70**

## Discussion

These results show that the CSF biomarkers of amyloid and tau metabolism were abnormal in HIV-infected patients with neurological diseases, but in a pattern that differed from Alzheimer's disease. This difference implies that different pathogenetic pathways underlie brain injury in ADC and Alzheimer's disease, and that the ADC 'signature' provided by these combined markers might help in diagnosis of HIV-related brain injury in untreated patients. The reduction of sAPP levels in both ADC and opportunistic infections also provides a clue to what may be an important component of altered neuronal metabolism in these conditions.

While the CSF biomarker profile has been adopted as a clinical tool for Alzheimer's disease and may even be useful in predicting incipient development of Alzheimer's disease in those with mild cognitive impairment [36], a similar approach has not been applied to the diagnosis of ADC/HIVE, at least formally [37]. In Alzheimer's disease, typical CSF changes include increases in t-tau and p-tau and a decrease in Aβ_1-42 _[36,38]. The diagnostic accuracy of these CSF biomarkers has been assessed in several studies, and their sensitivity and specificity in comparison to cognitively normal elderly people are each approximately 90 percent [39]. The availability of this objective, laboratory-based approach contrasts with the clinical syndromic basis of ADC/HIVE diagnosis [40]. While not supporting a pathogenetic similarity of ADC/HIVE and Alzheimer's disease or an Alzheimer's disease-like CSF biomarker pattern in ADC/HIVE, these results do suggest that amyloid and tau metabolism are perturbed in HIV-related brain injury, and that combined measurement of CSF biomarkers may have clinical application in certain settings.

### Amyloid metabolites

**Figure 4:**
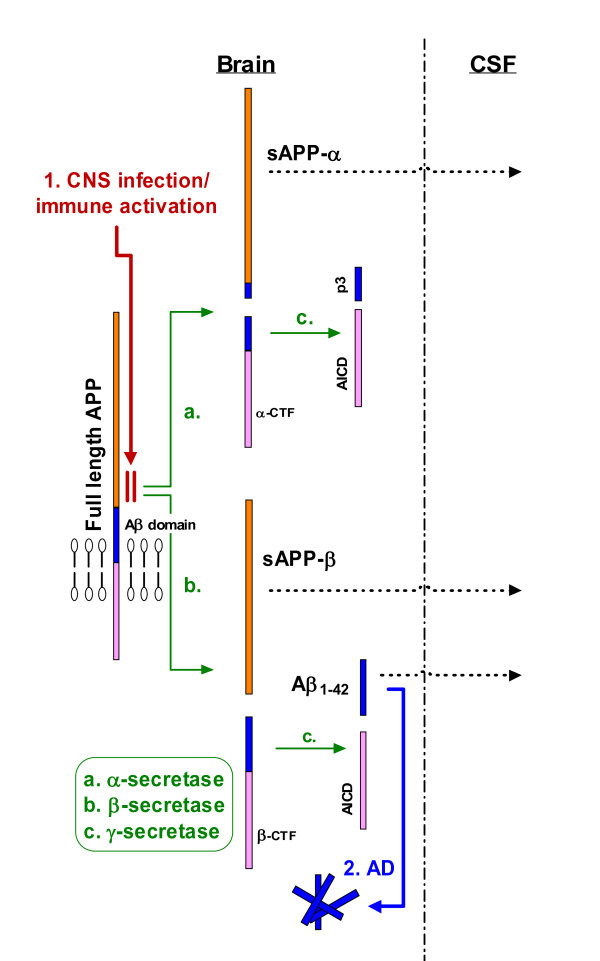
**Hypothesis of alterations in amyloid metabolism in ADC and opportunistic infections compared to AD**. The diagram shows proteolytic cleavages of the largest isoform of APP (APP770) and interprets the observed differences in effects of HIV and opportunistic infections from those of Alzheimer's disease on these pathways. In the non-amyloidogenic pathway (a.), cleavage by α-secretase occurs after residue 687, which enables the secretion of the large, soluble ectodomain of APP (sAPPα) into the medium and retention of the 83-residue C-terminal fragment (α-CTF) in the membrane. The α-CTF fragment can undergo cleavage by γ-secretase at residue 711 or 713 to release the p3 peptides (c.). In the amyloidogenic pathway (b.), β-secretase cleaves after residue 671, which causes the secretion of the slightly truncated sAPPβ molecule and the retention of a 99 residue C-terminal fragment (β-CTF). This fragment can undergo further cleavage by γ-secretase to release 40 or 42 aminoacid-long Aβ fragments (c.). Cleavage of both α- and β-CTF by γ-secretase releases the APP intracellular domain (AICD) into the cytoplasm. Hypothesized differences in the steps leading to CSF biomarker changes include: 1. an effect of CNS infection or immune activation on an early step in APP metabolism, in contrast to 2. deposition of Aβ_1-42 _in Alzheimer's disease[41].

APP is an ubiquitously expressed transmembrane protein that undergoes proteolytic processing by different secretases, resulting in generation of metabolites with varying potential pathological consequences (Figure 4) (for review, Andreasson et al [41]). Cleavage by α-secretase leads to production of soluble sAPPα that is shed from the cell membrane and can diffuse into the CSF where its concentration can be measured. This process has been referred to as the *non-amyloidogenic *pathway since it does not generate pathogenic Aβ. By contrast, cleavage of APP by β-secretase produces sAPPβ, also released and accessible to CSF for measurement, and β-CTF which, after further cleavage by γ-secretase, forms Aβ peptides, including Aβ_1-42_. In aggregated form, the latter is the major component of Alzheimer's disease plaques and has been proposed as one of the key molecules responsible for neurodegeneration in Alzheimer's disease [38]. CSF levels of sAPPα and sAPPβ are unaltered [42,43] or mildly elevated in sporadic Alzheimer's disease [44]. The reduced CSF Aβ_1-42 _concentration in Alzheimer's disease patients is speculated to result from deposition of aggregates that are then sequestered in brain, preventing 'normal' levels from reaching the ventriculo-subarachnoid space [45,46].

The mechanisms underlying the reduced CSF sAPPs in both ADC and the opportunistic infections are uncertain but may relate to the well-documented brain deposition of APP in both frank HIVE and in brains without identified foci of HIV infection [47]. The parallel reduction of sAPPs in ADC and opportunistic infections contrasts with the reduction of CSF Aβ_1-42 _in Alzheimer's disease which appears to be selective and independent of sAPPα or sAPPβ levels. The overall correlation between both sAPPs and Aβ_1-42 _was relatively weak, and the modest reduction of Aβ_1-42 _noted in some ADC patients and the opportunistic infections might be secondary to earlier inhibition of the APP pathway and limiting levels of the C-terminal fragment of β-cleaved APP available as substrate for further cleavage by γ-secretase rather than as a result of amyloid plaque deposition as in Alzheimer's disease. This is also consistent with pathological studies showing that Aβ-containing plaques characteristic of Alzheimer's disease are not a common feature in HIV [16,17].

It should be noted, however, that this remains a controversial issue [15]. Some studies report prevalence of 'diffuse' amyloid plaques, not fulfilling the CERAD neuropathological criteria for Alzheimer's disease [48] in AIDS patients [15,18,19,49]. Recently Achim and colleagues [50] reported abundant Aβ immunostaining in pyramidal neurons and along axonal tracts in brains of HIV-infected subjects. They found higher levels of intraneuronal Aβ immunoreactivity in HIVE than in HIV+ cases without this pathology. Double-labeling analysis showed that the Aβ-immunoreactive granules in neurons co-localized with lysosomal markers, and immunoelectronmicroscopy showed Aβ was often found in structures morphologically similar to autophagosomes. Most of these studies used the 4G8 antibody that does not distinguish between Aβ and full-length APP, though a second antibody, 82E1, that is more specific for Aβ was also tested and said to show similar results. The earlier reports by Nebuloni and colleagues [47] of accumulation of axonal APP in proximity to foci of HIVE have also been confirmed in a simian immunodeficiency virus (SIV) model of encephalitis [51]. It seems a reasonable hypothesis that the reduction of soluble forms of APP in CSF may be linked to the accumulation of APP in axons shown in these studies, perhaps through alteration in processing, transport or release of these forms into the extracellular fluid.

The finding of decreased CSF sAPPs not only in ADC but also in patients with various CNS opportunistic infections indicates that the cause is not specific to ADC/HIVE pathogenesis. Because immune activation, inflammation and cytokine production are common to ADC and CNS opportunistic infections, this broad category is a reasonable starting point for future study. It is curious that both meningitis without substantial brain parenchymal invasion (related to cryptococcal infection) and focal brain diseases (toxoplasmosis and PML) were capable of reducing CSF sAPPs, which presumably derive from throughout the brain. Additionally, we have found a marked transient reduction of CSF Aβ_1-42 _in bacterial meningitis [52], and also recently detected significant reductions in sAPPs and Aβ_1-42 _levels in multiple sclerosis [53], demonstrating that this is not confined to the context of HIV infection. This may all be taken as supporting a toxic effect of cytokines or other molecules diffusing from sites of inflammation to cause a generalized alteration in amyloid metabolism, though this needs further study. It will be important to more directly examine whether changes in sAPPs do indeed correlate with CNS immune activation or particular components of inflammatory reactions both in human diseases in vivo and in cell culture models that can be used to dissect cause and effect. A further important subject for exploration is whether these changes in amyloid metabolism are harmful or protective.

### Tau metabolites

CSF t-tau but not p-tau was elevated in some ADC patients and the opportunistic infection group. The overall impression of t-tau in this series is that both ADC/HIVE and opportunistic infections may increase t-tau leakage into the CSF, but not consistently. The relatively modest changes in t-tau levels in ADC, as compared to Alzheimer's disease, may relate to the predominating subcortical pathology of ADC/HIVE [54], since tau is expressed most prominently in non-myelinated cortical axons [55]. The inconsistency of t-tau elevations and the apparent limited sensitivity of t-tau to detect this type of injury likely also explain conflicts among previous reports.

CSF p-tau exhibited an even more consistent contrast between HIV CNS diseases and Alzheimer's disease, remaining at normal levels in the former and, as expected, elevated in the latter. The observations of normal levels of p-tau in all the HIV+ subjects are also consistent with the lack of neurofibrillary tangles in ADC/HIVE, one of the neuropathological hallmarks of Alzheimer's disease [16,56]. Thus, while increases in brain tau deposition have been reported in HIV infection, frank neuronal tangles are an uncommon and inconsistent finding [57,58]. This difference further supports the broader concept that ADC/HIVE and Alzheimer's disease do not share major mechanisms of neuronal injury.

### Application of neural biomarkers to diagnosis and patient management

The aggregate results of measurement of these neural CSF markers suggest that the pattern of alterations in amyloid- and tau-related markers may be useful in diagnosis of ADC in certain settings. ADC in the developed world now most frequently presents in patients who are not receiving antiretroviral therapy, often also have a high prevalence of background neurological abnormalities that can confuse ADC diagnosis [11] and contrasts with the 'pure forms' that allowed clinical definition of the condition earlier in the epidemic [5,59]. There is thus an increasing need for more objective, laboratory-based approaches to ADC/HIVE diagnosis to supplement or displace simple clinical recognition [40].

On the other hand, further studies will be needed before these findings can be extended to the problem of treated patients with cognitive dysfunction which now has moved to the foreground as a major issue of neurological diagnosis and treatment in the developed world [28]. The increased lifespan conferred by treatment has highlighted the importance of more subtle or indolent CNS injury related to early viral invasion, persistent subclinical infection and continued immune activation acting alone or in concert with other assaults on the CNS with age, including Alzheimer's disease [13-15]. This needs to be addressed directly by future studies focusing on these patients.

### Limitations

This study was limited by its retrospective, cross-sectional design and the use of a convenience sample. As different subject groups came from different centers, it couldn't be ruled out that they differed also in other aspects than diagnosis. To address some of the issues discussed here, particularly applicability to diagnosis, a prospective study would be useful. A longitudinal study might also test the use of these markers to predict the development of ADC in those without clear symptoms. The study also did not directly assess treated patients with milder cognitive dysfunction, nor did it include a substantial number of older HIV-infected subjects in the years most vulnerable to Alzheimer's disease. Finally, we did not assess HIV+ patients with known Alzheimer's disease, though we are not aware of reports of patients with this combination of conditions. Further studies are clearly needed before our findings of distinct changes in sAPPs, variable elevations in t-tau, and normal levels of p-tau can be applied diagnostically to treated patients with milder cognitive abnormalities [28].

## Conclusions

These results clearly show that ADC and Alzheimer's disease are characterized by distinct CSF tau and amyloid biomarker patterns suggesting distinct pathogenetic processes underlying the two conditions. The similar changes associated with ADC/HIVE and CNS opportunistic infections suggest that they likely relate to CNS immune activation and inflammation. While the underlying mechanism needs to be examined more directly in model systems and using other approaches, these observations suggest that the inflammation of HIV and other brain infections leads to abnormal APP processing or transport in neurons and axons, and that this may provide an indicator of an important component of CNS injury.

## Abbreviations

AD: Alzheimer's Disease; ADC: AIDS dementia complex; AICD: APP intracellular domain; ANI: asymptomatic neurocognitive impairment (associated with HIV infection); APP amyloid precursor protein; sAPPα and sAPPβ: the alpha and beta forms of soluble APP; Aβ: amyloid beta; AUC: area under the concentration curve; antiretroviral therapy: combination antiretroviral therapy; CMV-E: cytomegalovirus encephalitis; CNS: central nervous system; CSF: cerebrospinal fluid; α- and β CTF: alpha and beta C-terminal fragment; crypto: cryptococcal meningitis; HAND: HIV-associated neurocognitive disorder; HIV: human immunodeficiency virus (and here refers to type one); HIV+: HIV infected; HIV-: HIV uninfected; HIVE: HIV encephalitis; OIs: opportunistic infections; contr o: older HIV- controls; contr y: younger HIV- controls; MND: minor neurocognitive disorder (associated with HIV infection); NA: neuroasymptomatic; PCA: principal component analysis; PML: progressive multifocal leukoencephalopathy; ROC: receiver operator characteristic; p-tau: hyperphosphorylated tau; t-tau: total tau; toxo: *Toxoplasma gondii *encephalitis; WBC: white blood cell.

## Competing interests

The authors declare that they have no competing interests.

## Authors' contributions

MG, JK, KB, PC, SS, LH, RWP and HZ contributed CSF samples and subject evaluations. The study was planned and interpreted and the data reviewed by all of the authors. The manuscript was drafted by MG, JK, RWP and HZ, and reviewed and revised by all the authors. HZ and KB were responsible for all of the biochemical analyses. UA performed the multivariate PCA analysis, and MG and RWP performed the other analyses and prepared the figures. All authors read and approved the final manuscript.

## Pre-publication history

The pre-publication history for this paper can be accessed here:

http://www.biomedcentral.com/1471-2377/9/63/prepub
